# Investigation of the Effectiveness of an Algorithm as an Auxiliary Method in Intraoperative Consultations of Central Nervous System Tumors

**DOI:** 10.5146/tjpath.2024.13494

**Published:** 2025-01-31

**Authors:** Emel Cakir, Ismail Saygin, Ayten Livaoglu, Gizem Teoman, Zeynep Sagnak Yilmaz, Arife Cicek Malat, Muserref Muge Ustaoglu

**Affiliations:** Department of Pathology, Sancaktepe Martyr Prof. Dr. İlhan Varank Training and Research Hospital, İstanbul, Türkiye; Faculty of Medicine, Karadeniz Technical University, Trabzon, Türkiye; Faculty of Medicine, Karadeniz Technical University, Trabzon, Türkiye; Trabzon Kanuni Training and Research Hospital, Trabzon, Türkiye

**Keywords:** Algorithm, Central nervous system tumors, Intraoperative consultation, Neuropathology

## Abstract

***Objective: *** One of the most difficult areas in a surgical pathology practice is intraoperative consultation. In a previous study, we proposed an algorithm that provides a systematic approach to intraoperative consultation for central nervous system tumors. Our aim was to demonstrate the effectiveness of this algorithm.

***Material and Methods:*** 102 cases were selected from intraoperative consultation procedures performed at our institution between 2012 and 2020. The algorithm was tested by five observers. The observers examined the smears and frozen sections without the algorithm, and then with the algorithm.

***Results: *** The percentage change in the rate of correct diagnoses made by the four observers (O) increased after using the algorithm (O2: 8%, O3: 5%, O4: 8% and O5: 13%), but decreased for only one observer (O1) (5%). The most common error made by the four observers was “grading of glial tumors” (O1: 40%; O2: 23%; O4: 40% and O5: 27.5%), and this group of errors was mostly corrected by using the algorithm (O1: 33%; O2: 3.8%; O4: 23% and O5: 10%). For two observers (O2 and O5), a statistically significant change in diagnostic levels was observed after using the algorithm (p=0.024 and p=0.040; respectively). In addition, thanks to the use of the algorithm, a high degree of agreement was found between the observers’ diagnoses (77.7%, p<0.001).

***Conclusion:*** In the intraoperative consultation of central nervous system lesions, algorithms can help to increase the accuracy of the diagnosis and reduce interobserver variability. This study demonstrates that an algorithmic approach is an effective method for pathologists in intraoperative consultation procedures.

## INTRODUCTION

The purpose of the intraoperative consultation (IC) is to assess the adequacy of the specimen, to provide the surgeon with immediate information about the nature of the lesion, or to investigate the presence of a tumor at the surgical margins. A successful IC procedure requires an awareness of the pitfalls and limitations and an understanding of the key stages of the process.

IC of central nervous system (CNS) lesions requires correlation of clinical, radiologic, and histologic data. Lesions that occur in patients at a specific site, with a specific radiologic pattern, and in a specific age group significantly narrow the list of differential diagnoses. This increases the possibility of making a precise diagnosis with the data obtained in the pre-analysis.

In order to make the best possible contribution to the surgical procedure, the necessary pre-analytical phase (clear, precise and effective communication with the neurosurgeon, obtaining information about the patient’s clinical, radiological and intraoperative findings and submitting the correct and sufficient sample to the laboratory under appropriate conditions) and the analytical phase (assessment of the adequacy of the tissue, correct preparation of the smear – frozen sections and accurate microscopic examination) must have been carried out. All information about the importance of each of these steps and how they should be performed can be found in the previous study (1).

The best way to make the correct diagnosis during the IC procedure is to first determine the tumor type. For this purpose, it is important to look at the tumor background, especially in smear slides, and try to identify one of the four main tumor types (glial, mesenchymal, neuronal and epithelial) based on certain characteristics (1). To perform the correct microscopic examination in the analytical phase, some previous studies have shown that smear preparations are important for determining cytologic features and frozen sections are important for determining structural features and that these two methods should be used together (1–5).

**Figure 1:**
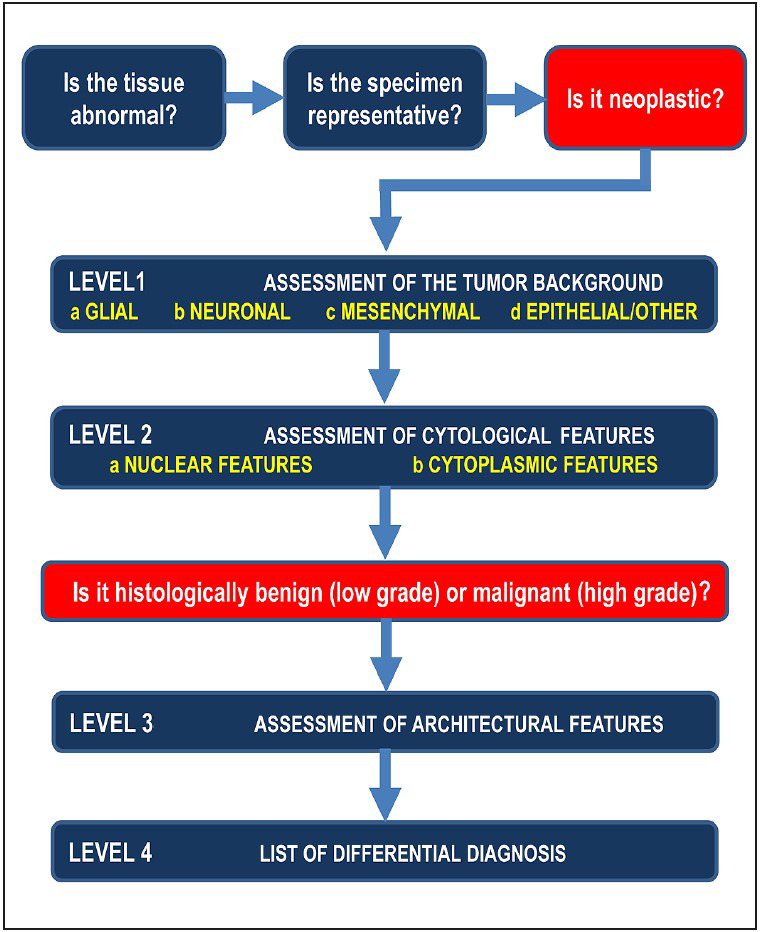
The algorithm of intraoperative consultation specimens for CNS tumors (1).

The accurate examination of smears and frozen sections prepared in the analytical phase and reaching the correct differential diagnosis may be possible by using an algorithm that helps the pathologist to combine all relevant information. To this end, in a previous study, we proposed an algorithm that the pathologist interprets by systematically evaluating the microscopic features with a series of standard questions (Figure 1).

In the post-analytic phase, the crucial role of the surgical pathologist is to report the IC diagnosis accurately and promptly.

The aim of this study is to test the “algorithm” developed to contribute to an accurate microscopic examination in the analytical phase. It also aims to determine whether it is useful in increasing both the rate of correct diagnoses and consistency between observers among pathologists working independently and in different environments.

## MATERIAL and METHODS

### Study Design

From the IC procedures performed at our institution between 2012-2020, 102 demonstrative cases suitable for evaluation were selected. First, the clinical and radiologic information of all cases, other initial IC diagnoses, and final diagnoses were reviewed by two authors (EÇ, İS). To determine the practical utility of the algorithm, it was tested by five observers who had no training in neuropathology and consisted of senior residents and general surgical pathologists working in two different institutions. To this end, the observers, who had the clinical/demographic features and radiologic information (radiology reports) available at the time of the original IC, re-examined the smears (one or more slides) and frozen sections of 102 cases, first without the algorithm, then with the algorithm. At the second examination with the algorithm, each observer was asked to use a checklist of algorithmic steps (1) and make the diagnosis. The diagnoses were then compared with the original IC diagnoses and the final diagnoses.

In the steps of the algorithm, the levels were defined as follows: Level 0: Only if the lesion can be defined as “neoplastic”; Level 1: If the tumor type can be determined; Level 2-3: If the tumor grade can be determined based on cytological + structural features, and Level 4: If a differential diagnosis list can be established (specific diagnosis with clinical and radiological findings) (1).

To test the effectiveness of the algorithm, first without the algorithm, then with the algorithm: a) the rates of correct diagnoses, b) the rates of incorrect diagnoses, c) diagnostic levels were determined. In addition, the agreement between the observers in the diagnoses obtained without and with the help of the algorithm was evaluated.

### Statistical Analysis

The statistical analyses were performed with the SPSS 23.0 package program. In the applications with and without the algorithm, the descriptive statistics are presented with numbers and percentages. The agreements between the observers are indicated with the intraclass correlation coefficient and confidence intervals. The McNemar-Bowker test was used to analyze the differences between the ratios of the categorical variables in the addicted groups. P-values of less than 0.05 were considered statistically significant.

## RESULTS

In the distribution of all IC cases between 2012 and 2020, the most common tumors are morphologically high-grade gliomas (37 cases; 36.2%), metastatic carcinomas (15 cases, 14.7%), low-grade gliomas (13 cases; 12.7%), meningiomas (10 cases; %). 9.8), ependymomas (9 cases, 8.8%), non-neoplastic lesions (6 cases, 5.8%), embryonal tumors (4 cases, 3.9%) and schwannomas (3 cases, 2.9%). In addition to the main case groups mentioned, there are 5 more diagnostic groups, each with 1 case. These are 1 hemangioblastoma, 1 melanocytoma, 1 craniopharyngioma, 1 neurocytoma and 1 lymphoma case.

The diagnostic level, misdiagnosis rate and accurate diagnosis rate of each observer in the smears and frozen sections of the 102 cases that they examined first without the algorithm and then with the algorithm are shown in (Table 1).

**Table 1:** Diagnostic level, misdiagnosis and accurate diagnosis rates achieved by observers with and without the algorithm

	**Observer 1**		**Observer 2**			**Observer 3**		**Observer 4**		**Observer 5**	
	**Algorithm**		**Algorithm**			**Algorithm**		**Algorithm**		**Algorithm**	
	**-**	**+**	**-**		**+**	**-**	**+**	**-**	**+**	**-**	**+**
No diagnosis(%)	2	2	0		0	4	2	2	2	0	0
**Diagnostic Level**											
Level 0 (%)	1	2	13		6	8	8	2	3	3	4
Level 1 (%)	15	19	18		11	40	27	28	25	38	28
Level 2-3 (%)	38	34	40		39	30	37	47	41	38	35
Level 4 (%)	44	43	29		44	18	26	21	29	21	33
**Misdiagnosis**											
Tumor degree + type (%)	1	2	5		5	7	5	2	3	3	3
Tumor degree (%)	8	9	10		5	5	9	12	5	10	4
Tumor type (%)	7	10	6		8	12	11	13	10	25	20
Specific diagnosis (%)	0	0	6		1	2	0	3	2	4	2
**Accurate Diagnosis (%)**	82	77	73		81	70	75	70	78	58	71

The correct diagnosis rates of the observers before and after using the algorithm are as follows: O1: from 82% to 77%, O2: from 75% to 83%, O3: from 73% to 78%, O4: from 69% to % to 77 and O5: from 58% to 71%. (Table 2)

**Table 2:** The correct diagnosis rates of the observers before and after using the algorithm

	**Observer 1**		**Observer 2**		**Observer 3**		**Observer 4**		**Observer 5**	
	**Algorithm**		**Algorithm**		**Algorithm**		**Algorithm**		**Algorithm**	
	-	+	-	+	-	+	-	+	-	+
The correct diagnosis rates (%)	82	77	75	83	73	78	69	77	58	71

The rate of “correct diagnosis” increased for four observers, with a maximum of 13% (rates of increase: O2: 8%, O3: 5%, O4: 8% and O5: 13%). The rate of “correct diagnosis” of only one observer (O1) decreased with the use of the algorithm (decrease of 5% from 82% to 77%).

For only one observer (O3), the rate of “defer to permanent sections” decreased from 4% to 2% with the algorithm. There were no changes for the others.

Grading of glial tumors was the most frequent error in four observers (errors before the algorithm: O1: 40%; O2: 23%; O4: 40% and O5: 27.5%); the rate of correct diagnosis with the algorithm was also highest in this group (grading of glial tumors) (errors after the algorithm: O1: 33%; O2: 3.8%; O4: 23% and O5: 10%). O3, who mainly misdiagnosed metastatic tumors without using the algorithm, had the highest rate of correct diagnoses in this group with the help of the algorithm (error: from 33% to 12.5%).

Other tumors that were misdiagnosed were metastatic tumors, ependymomas or meningiomas.

Six cases were misdiagnosed by all observers: 2 were carcinoma metastases, 1 glioblastoma, 1 ependymoma with anaplastic areas, 1 neuroglial tissue (non-tumoral) and 1 lymphoma.

The diagnostic levels that the observers were able to achieve during the intraoperative consultations are shown in (Table 3, Table 4, Table 5, Table 6, Table 7). For cases such as metastases, lymphomas, melanomas, etc., diagnostic level 2-3 were not applicable. In these cases, observers were expected to make a diagnosis at level 0, level 1 or level 4. According to the algorithm, four observers showed a decrease in level 0 and 1 diagnoses (decrease: O2: 14.2%, O3: 14.2%, O4: 1.9% and O5: 8.8%), while there was an increase in level 2-3 and 4 diagnoses (increase: O2: 14.2%, O3: 15.6%, O4: 1.9% and O5: 8.8%). In contrast, only one observer (O1) showed an increase in diagnoses at levels 0 and 1 after using the algorithm, while diagnoses at levels 2-3 and 4 decreased. A statistically significant change was observed in O2 and O5 after the algorithm (p=0.024 and p=0.040, respectively).

**Table 3:** Diagnostic levels that could be reached by **Observer-1** during intraoperative consultations

		**With the algorithm**					
		**No diagnosis**	**Level 0**	**Level 1**	**Level 2-3**	**Level 4**	**Total**
Without the algorithm	No diagnosis	2	0	0	0	0	2
	Level 0	0	1	0	0	0	1
	Level 1	0	1	12	1	1	15
	Level 2-3	0	0	2	34	3	39
	Level 4	0	0	5	0	40	45
	Total	2	2	19	35	44	102

P=0.136

**Table 4:** Diagnostic levels that could be reached by **Observer-2 **during intraoperative consultations

		**With the algorithm**					
		**No diagnosis**	**Level 0**	**Level 1**	**Level 2-3**	**Level 4**	**Total**
Without the algorithm	No diagnosis	0	0	0	0	0	0
	Level 0	0	5	1	4	3	13
	Level 1	0	0	7	7	4	18
	Level 2-3	0	1	3	23	14	41
	Level 4	0	0	0	6	24	30
	Total	0	6	11	40	45	102

P=0.024

**Table 5:** Diagnostic levels that could be reached by **Observer-3 **during intraoperative consultations

		**With the algorithm**					
		**No diagnosis**	**Level 0**	**Level 1**	**Level 2-3**	**Level 4**	**Total**
Without the algorithm	No diagnosis	0	2	0	0	2	4
	Level 0	1	4	2	0	1	8
	Level 1	1	0	19	15	6	41
	Level 2-3	0	1	5	19	6	31
	Level 4	0	1	1	4	12	18
	Total	2	8	27	38	27	102

P=0.083

**Table 6:** Diagnostic levels that could be reached by **Observer-4 **during intraoperative consultations

		**With the algorithm**					
		**No diagnosis**	**Level 0**	**Level 1**	**Level 2-3**	**Level 4**	**Total**
Without the algorithm	No diagnosis	1	0	1	0	0	2
	Level 0	0	2	0	0	0	2
	Level 1	0	0	13	7	9	29
	Level 2-3	0	0	9	33	6	48
	Level 4	1	1	3	2	14	21
	Total	2	3	26	42	29	102

P=0.220

**Table 7:** Diagnostic levels that could be reached by **Observer-5 **during intraoperative consultations

		**With the algorithm**					
		**No diagnosis**	**Level 0**	**Level 1**	**Level 2-3**	**Level 4**	**Total**
Without the algorithm	No diagnosis	0	0	0	0	0	0
	Level 0	0	2	1	0	0	3
	Level 1	0	1	20	8	10	39
	Level 2-3	0	1	8	26	4	39
	Level 4	0	0	0	2	19	21
	Total	0	4	29	36	33	102

P=0.040

It was observed that the interobserver agreement increased when the slides were examined using the algorithm (from 68.2% to 77.7%). This is because for all observers except O1, both the rate of correct diagnoses and the diagnostic levels increased after the algorithm. However, interobserver consistency remained at the same level. Because the observers made some errors despite using the algorithm. Some of these were errors that had already been identified during the initial examination and some were other errors. It was found that the algorithm increased the diagnostic accuracy rates in different tumor groups in different observers. This suggests that the algorithm contributed differently for observers with different levels of knowledge. The interobserver consistency was statistically significant both in the examination with and without the algorithm (Table 8).

**Table 8:** Interobserver consistency

**Interobserver (level)**	**Intraclass Correlation**	
	**95%C.I.**	**p value**
Without the algorithm	68.2 (57.3-77.0)	<0.001
With the algorithm	77.7 (70.0-83.9)	<0.001
**Interobserver (misdiagnosis)**		
Without the algorithm	70.2 (60.0-78.5)	<0.001
With the algorithm	70.1 (59.8-78.4)	<0.001

## DISCUSSION

Few studies on IC and definitive diagnoses have pointed out the difficulties in accurately diagnosing tumors (4,6–9). Some of these difficulties include distinguishing diffuse gliomas from reactive or inflammatory lesions, differentiating spindle cell tumors (e.g., meningioma versus schwannoma), differentiating primary tumors from metastatic tumors, or determining the grade of meningothelial / glial tumors. In addition, the differential diagnosis of astrocytomas and oligodendrogliomas as well as infiltrative and well-circumscribed low-grade glial tumors has always been difficult (4,7,10–12).

Modi et al. reported in their study that most discrepancies between IC and permanent diagnoses occurred in astrocytomas, ependymomas, metastatic tumors, meningiomas and rhabdoid tumors (13,14).

In the study by Plesec and Prayson, <3% mismatch was found in 2156 cases over a period of 8 years (7). According to this study, approximately 80% of the inconsistent cases were spindle cell lesions, astrocytoma versus oligodendroglioma, lymphoma, reactive processes versus neoplastic processes, and tumor grading errors (7).

Geramizadeh et al. found that the most frequent inconsistency occurred in CNS tumors (14 cases). Ten inconsistent cases (71.3%) in the CNS were ‘spindle cell tumors’, in which a distinction must be made between meningiomas and schwannomas (15). Another common discordance in this study was between low-grade gliomas and reactive gliosis (3 cases; 21.3%).

We also had similar problems in our study. Four of the five observers most frequently erred in the ‘grading of glial tumors’ and second most often in metastatic tumors. Metastatic tumors were most frequently confused with high-grade glial tumors, followed by meningiomas. Meanwhile, ependymomas were most commonly confused with other glial tumors, followed by mesenchymal tumors. In addition, meningiomas were most commonly confused with schwannomas.

However, thanks to the algorithm, these four observers showed a significant improvement in the grading of glial tumors, the most common error they made (O1: from 40% to 33%; O2: from 23% to 3.8%; O4: from 40% to 23%; O5: from 27.5% to 10%). The other observer also achieved the best improvement in her most common error (metastatic tumors) (error: from 33% to 12.5%).

When investigating the six cases that were misdiagnosed by all observers, it was found that one of the two carcinoma metastases and one ependymoma case with anaplastic areas were also misdiagnosed by the reference author who diagnosed the cases and signed the original reports.

Some of the diagnostic errors were due to lack of experience, while a significant part was due to technical reasons. These were sampling features (small tissue, partial representation of lesion characteristics) and freezing artifacts that occurred during the IC process. These technical difficulties also made it difficult for observers to recognize the lesion. For this reason, in our previous article (1) we emphasized what needs to be done in the pre-analytical phase. Improvement in this phase is one of the most important steps to make the correct diagnosis. This is because even if the algorithm is perfect, errors in tissue preparation could prevent the algorithm from being used.

The algorithm used during the IC procedure provided a standard checklist to follow and helped pathologists consider options that they may not have initially considered during the differential diagnosis. Thanks to the interpretation of the background in the smears, it was possible to start with a narrower list of differential diagnoses. Additionally, the algorithm aided in better recognition of both low- and high-grade features within the same tumor type. In this way, a descriptive diagnosis/category was obtained, which is a more appropriate diagnosis to be made during the surgical procedure.

The algorithm enabled the observers to increase their diagnostic rates at the advanced levels (levels 2-3 and 4) and consequently empowered them, so that they could provide the neurosurgeon with more specific treatment recommendations.

The situation of O1differs from the others in every respect. The rate of correct diagnoses of O1 decreased with the usage of the algorithm. It turned out that this observer could not benefit from the algorithm for any error. In addition, there was an increase in level 0 and 1 diagnoses for this observer after the algorithm, while a decrease was observed for level 2-3 and 4 diagnoses. O1, who was not helped by the algorithm, was the observer with the highest rate of correct diagnoses without using the algorithm. In contrast, the observer who had the lowest rate of correct diagnoses without the algorithm (O5) had the highest rate of correct diagnoses after using the algorithm. That is, observers with less basic knowledge showed the best improvement, while observers with better basic knowledge benefited less from the algorithm. These results made us think that the effectiveness of this algorithm might vary among pathologists with different levels of knowledge. At the same time, these observers who lack neuropathological practice, even if they are better at first sight, must have the basic knowledge that we use in IC and that we try to explain step by step in the algorithm (how to recognize the background of tumors, cytological and structural features of each tumor type -glial, mesenchymal, neuronal and epithelial). Otherwise, it will not be possible to use the algorithm effectively. For this reason, we plan to create a guide that contains this basic information as well as the algorithm.

The most limiting factor of the study is the imbalance representation of tumor diversity. Since the majority of the material sent for IC consisted of glial tumors, the study results were noticeably affected, with the highest degree of error found in grading of glial tumors. However, these disease rates are still encountered in everyday working life and reflect our daily practice. In addition, another noteworthy limitation is the lack of opportunity for observers to contact the doctor and check the radiological findings. Providing this information in daily practice would increase accuracy rates.

In summary, the algorithm has the potential to assist surgical pathologists to perform IC of CNS tumors. Accordingly, it will be possible to reduce the rate of misdiagnosis that occurs during IC of CNS tumors. Therefore, the best contribution could be made to the patient’s prognosis by offering appropriate guidance on whether to proceed with or cease the operation.

## Ethics Committee Approval

This study was approved by the Karadeniz Technical University Ethics Committee with protocol number 2021/282.

## Conflict of Interest

The authors declare that they have no conflicts of interest.
